# Labour and premature delivery differentially affect the expression of the endocannabinoid system in the human placenta

**DOI:** 10.1007/s00418-023-02236-y

**Published:** 2023-09-26

**Authors:** Anthony H. Taylor, Panos Bachkangi, Justin C. Konje

**Affiliations:** 1 Department of Cancer Studies and Molecular Medicine, Endocannabinoid Research Group, Reproductive Sciences Section, University of Leicester, Leicester, UK; 2 Department of Molecular and Cell Biology, University of Leicester, Leicester, UK; 3 Department of Obstetrics and Gynaecology, Queen’s Hospital, University Hospitals of Derby and Burton NHS Foundation Trust, Burton On Trent, UK; 4 Feto Maternal Centre, Al Markhiya, Doha, Qatar

**Keywords:** Cannabinoid receptor, Fatty acid amide hydrolase, Endocannabinoid, Labour, Placenta, Prematurity, Trophoblast

## Abstract

Plasma concentrations of *N*-arachidonyletholamine (AEA), *N*-oleoylethanolamide (OEA) and *N*-palmitoylethanolamide (PEA) increase at term and can predict when a woman is likely to go into labour. We hypothesised that increased plasma AEA concentrations in women in preterm and term labour might also be increased and have a function in the placenta at the end of pregnancy. Here we examined the expression of the *N*-acylethanolamine-modulating enzymes fatty acid amide hydrolase (FAAH) and *N*-acyl-phosphatidylethanolamine-specific phospholipase-D (NAPE-PLD) and of the cannabinoid receptors (CB1 and CB2) in the placenta and their activation in an in vitro model of the third-trimester placenta to determine if those expressions change with labour and have functional significance. Expression of CB1, CB2, FAAH and NAPE-PLD was examined by immunohistochemistry (IHC) and RT-qPCR in placental samples obtained from four patient groups: preterm not in labour (PTNL), term not in labour (TNL), preterm in labour (PTL) and term in labour (TL). Additionally, the effects of AEA on a third-trimester human cell line (TCL-1) were evaluated. All ECS components were present in the third-trimester placenta, with NAPE-PLD and CB2 being the key modulated proteins in terms of expression. Functionally, AEA reduced TCL-1 cell numbers through the actions of the CB2 receptor whilst CB1 maintained placental integrity through the expression of the transcription regulators histone deacetylase 3, thyroid hormone receptor β 1 and the modulation of 5α reductase type 1. The placenta in the third trimester and at term is different from the placenta in the first trimester with respect to the expression of CB1, CB2, FAAH and NAPE-PLD, and the expression of these proteins is affected by labour. These data suggest that early perturbation of some ECS components in the placenta may cause AEA-induced PTL and thus PTB.

## Introduction

Preterm birth (PTB) remains one the most common obstetric complications affecting anywhere between 5% and 18% of pregnancies worldwide that results in significant morbidity and mortality (Bachkangi et al. 2019b; World Health Organization 2022; Tucker and McGuire 2004). The exact aetiopathogenesis of PTB is not fully understood, and although many factors have been shown to be associated with its initiation and progression, the precise mechanism leading to its initiation may never be known as it is likely to be multifactorial (Goldenberg et al. 2008). In this regard, biomarkers of impending birth, especially those that predict when clinical labour may occur, have been sought (Sharabi-Nov et al. 2020; Zhao et al. 2020).

We have previously demonstrated that the levels the endocannabinoid anandamide (AEA) are significantly elevated in the plasma of women at term and increase further when they go into spontaneous labour or deliver preterm (Habayeb et al. 2008a; Nallendran et al. 2010). Indeed, we have also examined the plasma concentrations of two *N*-acyl congeners (*N*-oleoylethanolamine and *N*-palmitoylethanolamine) and shown that the plasma concentrations of these two lipids are not only significantly increased at term, but are also increased in women in preterm labour and can be used as a predictive test to determine when labour may occur in asymptomatic women (Bachkangi et al. 2019a).

Exactly how these *N*-acylethanolamines (NAEs) act on pregnancy-associated tissues is currently unknown, although we and others have previously shown that these lipids (a) act on the placenta in early pregnancy (Habayeb et al. 2004b, 2008a, b; Maia et al. 2020b; Melford et al. 2014; Sood et al. 2006; Taylor et al. 2007, 2010b; Trabucco et al. 2009) and (b) play a role in pregnancy maintenance during early pregnancy (Trabucco et al. 2009) and (c) that their dysregulation is associated with threatened and spontaneous miscarriage (Karasu et al. 2011; Taylor et al. 2010b, 2011a). This is because placental tissues express the cannabinoid receptors 1 (CB1) and 2 (CB2) to which the *N*-acylethanolamines bind and activate, with their loss resulting in placental insufficiency (Habayeb et al. 2004b; Maia et al. 2020b). This goes partway to explaining the detrimental effects of cannabis use during pregnancy on the placenta since it disrupts normal endocannabinoid system (ECS) signalling (Habayeb et al. 2008a; Maia et al. 2019, 2020a). As a result, there are also changes in the expression of the *N*-acylethanolamine regulatory enzymes, fatty acid amide hydrolase** (**FAAH), which is involved in the degradation of ligands, and *N*-acyl phosphatidylethanolamine-specific phospholipase-D (NAPE-PLD) (Habayeb et al. 2008a; Kenney et al. 1999; Park et al. 2003; Trabucco et al. 2009), which is the enzyme that regulates the rate-limiting step in the production of the *N*-acylethanolamines, especially anandamide. This work is summarised in a recent review (Kozakiewicz et al. 2021).

Since increased plasma NAE concentrations and disruption of ECS function in the first-trimester placenta are both associated with threatened and spontaneous miscarriage (Bachkangi et al. 2019a; Fonseca et al. 2012; Habayeb et al. 2004b, 2004c, 2008a, 2008b; Karasu et al. 2011, 2014; Kozakiewicz et al. 2021; Maia et al. 2020b; Melford et al. 2014; Nallendran et al. 2010; Park et al. 2003; Sun et al. 2016; Taylor et al. 2007, 2010b; Trabucco et al. 2009), and similar changes occur during parturition (Ayakannu et al. 2019; Brighton et al. 2009) without affecting the contraction of the myometrium where AEA causes relaxation (Ayakannu et al. 2019; Brighton et al. 2009), we hypothesised that the increased plasma AEA concentrations that are associated with parturition could also result in the dysregulation of the placental ECS at the end of pregnancy and in labour (Schuel and Burkman 2006). The aim of the present study was thus to examine the expression of the cannabinoid receptors and the NAE-modulating enzymes in the transition from the quiescent pregnant state to the point of active labour. Additionally, the expression of these proteins in preterm labour was examined to see if the expression patterns differed from those of term labour and as such could be used as a marker of impending preterm labour.

Previously, we have also demonstrated that first-trimester trophoblast cells (BeWo) respond to the phytocannabinoid Δ^9^-THC by increasing their expression of many genes, including (histone deacetylase 3 (HDAC3), and decreasing others such as thyroid receptor β 1 (TRβ1) and 5α reductase type 1 (5αR1; (Khare et al. 2006)), with AEA increasing the expression of HDAC3, whilst also decreasing that of TRβ1 and 5αR1 (Taylor et al. 2009a). HDAC3, TRβ1 and 5αR1 have several important functions in the placenta, including stabilisation of the syncytiotrophoblast through syncytin protein expression (Chuang et al. 2006), maintaining the early trophoblast and thyroid hormone transport (Ohara et al. 2004) and averting foetal death through prevention of androgen toxicity (Mahendroo et al. 1997), respectively. In our previous experiments. BeWo cells simultaneously underwent decreased cell growth in response to both Δ^9^-THC and AEA. Hence, we hypothesised that AEA would do the same in the third-trimester trophoblast cell line TCL-1; therefore, this hypothesis was also tested.

## Materials and methods

### Patients and sample procurement

All patients were recruited from either the Prematurity Prevention Clinic or Obstetric Labour Wards (emergency admissions) of the Department of Obstetrics & Gynaecology, Leicester Royal Infirmary at the University Hospitals of Leicester NHS Trust. All participants provided informed consent, and the study was approved by the Leicestershire, Northamptonshire and Rutland Research Ethics Committee (reference number 06/Q2501/48). Women with multiple pregnancies, or medical conditions (e.g. hypertensive disorders of pregnancy, gestational diabetes, connective tissue disease), or with suspected chorioamnionitis and/or placental abruption were excluded. A power analysis of similar studies (Taylor et al. 2009b) had shown that six samples were sufficient for each group to demonstrate a 40% change in gene expression with *α* = 0.05 and *β* = 0.80. However, previous experience indicated that insufficient or incomplete samples (e.g. chorion without amnion in foetal membrane samples or calcification of the placental cotyledons) sometimes occurred, and so a collection of ten samples (minimum) for each group was made from which six good and representative samples were selected. Placental biopsies were obtained from four patient groups. The first were women who were preterm and not in labour (PTNL) and acted as the control group. These were those women having a caesarean section for foetal reasons (e.g. severe intrauterine growth restriction), or maternal obstetric complications (e.g. severe pre-eclampsia) and who were not in active or suspected labour. The second group were women at term (at least 37 completed weeks of gestation) and not in labour (TNL) having elective caesarean section. The third group were preterm (< 37 completed weeks of gestation) and in active labour and who had a vaginal delivery (PTL). The fourth group were women at term and in active labour (TL) and who delivered vaginally. A blood sample for plasma AEA measurement was collected from each woman on the same day as the placental tissue.

### Plasma AEA measurement

The processing, storage and measurement of plasma AEA was performed as previously described using tandem ultra high performance liquid chromatography-tandem mass spectrometry (UHPLC-MS/MS, Marczylo et al. 2009). Every woman included in this study also had plasma NAE concentrations measured; these data were included in our previous publication (Bachkangi et al. 2019a).

### Tissue collection

For each woman, the placenta was collected from the delivery suite and transported on ice to the research laboratory where a large cotyledon was selected and dissected free under sterile conditions. The piece was then divided into two parts: one for histology/immunohistochemistry (IHC) and the other for RNA extraction. Biopsies measuring 1 cm × 1 cm × 0.5 cm from these cotyledons were placed into a tissue cassette and fixed in formalin (10% formaldehyde in normal saline) for 48 h, with the formalin changed at 24 h. Samples were then dehydrated and embedded in paraffin wax and, after air-drying at 37 °C for 1 week, stored at room temperature, until all samples from the four groups had been collected. At this point, the tissues were cut with a microtome into 4-μm-thick sections, mounted on silanised microscope slides and re-dried at 37 °C for 7 days. Standard haematoxylin and eosin staining of sections was used to ensure sufficient, good-quality tissue was available for IHC. Six placenta biopsies from each group with histologically similar appearances were selected for IHC. Additionally, a portion of placenta weighing approximately 100 mg was placed in a sterile polypropylene tube, and the tissue was snap-frozen in liquid nitrogen for up to 5 min. The frozen tissue was then stored at −80 °C for future RNA preparation and transcript analyses.

### RNA preparation

RNA preparation was based on a method we previously optimised (Habayeb et al. 2008a). Briefly, the RNA extraction was performed using TRIzol reagent (Invitrogen, Paisley, UK) as per the manufacturer instructions, and then treated with RNase-free DNase 1 in the presence of the RNase inhibitor RNasin (both from Promega Corp, Southampton, UK) using the manufacturer’s instructions. The RNA was re-dissolved in 100 μl of DEPC-treated water by heating at 56 °C for 5 min and stored at –80 °C.

### RT-qPCR

The mRNA was converted to cDNA using AMV-RT, as described (Kenney et al. 1999). Real-time quantitative PCR (qPCR) was then performed using previously validated SyBr methods (Park et al. 2003), in a Roche Lightcycler (Habayeb et al. 2008a) using the primers and annealing temperatures listed in Table 1. The levels of each gene’s transcript were evaluated using the relative expression method of Livak and Schmittgen (Livak and Schmittgen 2001) and confirmed using REST2008 software (Pfaffl et al. 2002) with GAPDH (Sood et al. 2006) as the normaliser gene, since GAPDH is not regulated in the placenta (Cleal et al. 2009).

**Table 1:** Primer sequences used in qPCR together with the annealing temperature and amplicon size

Primer name	Sequence (5′ to 3′)*	Temperature (°C)	Size (bp)
5α reductase type 1F	TGGCGCTTCTCTATGGACTT	60	369
5α reductase type 1R	GGAAGCAACACTGCAGTTGA		
CB1F	CTTCCCACAGAAATTCCC	62.4	853
CB1R	TACCTTCCCATCCTCAGA		
CB2F	GGCCGTCAGCTACACTATGC	62.4	851
CB2R	ATCTCGGGGCTTCTTCTTTT		
NAPE-PLDF	AAGAGATAGGAAAAAGATTTGGACCTT	60	99
NAPE-PLDR	CTGGGTCTACATGCTGGTATTTCA		
FAAHF	GGCCGTCAGCTACACTATGC	59	59
FAAHR	ATCAGTCGCTCCACCTCCC		
GAPDHF	AGAACATCATCCCTGCCTC	60	347
GAPDHR	GCCAAATTCGTTGTCATACC		
HDAC3F	GGGACATTATTGGCAGTG	55	233
HDAC3R	GGATTCAGGTGTTAGGGAG		
TRβ 1F	GAGATTTCCTCCTGGTTG	55	314
TRβ 1R	AGTGCTTCGGTTTGTCCC		

*All sequences are 5′–3′; *F* forward primer, *R* reverse primer

### Immunohistochemistry

The antibodies used for immunohistochemistry (IHC) were all from commercial sources. IHC for CB1 (rabbit polyclonal, Sigma C1108, Sigma-Aldrich, Poole, Dorset, UK; 1:500 dilution), CB2 (rabbit polyclonal, Sigma C1358, 1:150 dilution) and FAAH (immune rabbit serum, Alpha Diagnostics International, Austin, TX, USA; FAAH11-S; 1:2000 dilution) were performed as described (Habayeb et al. 2008a), using rabbit isotype IgG (CB1 and CB2) or non-immunised rabbit serum (FAAH) as the antibody negative control. The NAPE-PLD (rabbit polyclonal, Sigma HPA024338; 1:50 dilution) IHC protocol was similar of that for CB1 and CB2 with some modifications. Briefly, after dewaxing and rehydration with water through graded ethanol solutions, endogenous peroxidase activity was quenched using H_2_O_2_ (3% v/v in ice-cold water) for 15 min and then washed in Tris-buffered saline (TBS) containing 10% Tween_20_ (TBS-Tween_20_). The NAPE-PLD primary antibody was prepared at a concentration of 1:50 in blocking solution (5% normal goat serum in TBS). For the negative controls, rabbit IgG at 1:50 diluted in blocking solution, was added. All slides were then incubated in a humid chamber at room temperature for 18 h. Primary antibody or IgG was removed by washing three times in TBS-Tween_20_ for 5 min. Biotinylated goat anti-rabbit IgG (diluted 1:400 in TBS) was added for 30 min. After washing in TBS, ABC Elite (Vector Laboratories, Peterborough, Cambridgeshire, UK) was added and incubated at room temperature for 30 min. Antibody–antigen complexes were visualised with 3, 3′-diaminobenzadine (DAB, Vector Labs) for 5 min. The slides were then washed in distilled water for 5 min and lightly counterstained in Mayer’s haematoxylin for 2 min, blued under running tap water for 5 min, dehydrated in 95% and 99% industrial methylated spirits (IMS), cleared twice in xylene, mounted using XAM mountant (BDH, Leicester, UK) and allowed to air-dry for 24 h before being viewed and subjected to histomorphometric analysis.

### Histomorphometric analysis

Histomorphometric analysis (H-score) of immunoreactive protein expression was performed as previously described (Taylor et al. 2010a). Briefly, stained tissue sections were examined on an Axioplan transmission microscope (Carl Zeiss, Welwyn Garden City, Herts, UK) at 200× (Plan-Neofluor 20× objective, NA 0.50) magnification and captured on a Sony DXC-151P 2/3 inch CCD camera mapping to 768 × 493 pixels (Sony Corp., Kanagawa-Ken, Japan). Sections were imaged and captured in the presence of daylight and medium neutral density filters with the lamp set at 6400 K. The output.zvi files were converted to .tif images, and where colour casts were obvious, the images were uniformly corrected to a neutral density background using Colorpilot version 5.4.0 (Colorpilot.com). Images were then analysed using image analysis software (ImageScope version 10.2.2.2319; Aperio Technologies, Inc., Vista, CA). The H-score values were determined independently for ten random fields per section, and the H-scores were combined to provide an overall average H-score for the entire tissue (Taylor et al. 2010a).

### Effects of AEA on TCL-1 cells

The immortalised third-trimester trophoblast cell line TCL-1 (Lewis et al. 1996) were maintained as a monolayer culture in RPMI 1640 (Flow Laboratories) supplemented with 10% mycoplasma screened and heat-inactivated foetal calf serum (FCS), l-glutamine (2 mM), penicillin (100 IU/ml) and streptomycin (100 IU/ml) (all from Gibco BRL, Inchinnan, Renfrewshire, Scotland). The cells were sub-passaged at 60% confluency (usually every 5–7 days). For experimental purposes the cells were cultured in RPMI 1640 medium with 10% FCS for an initial 24 h to allow complete adherence to the substratum. AEA (Cayman Chemicals, Cambridge Bioscience, Cambridge, Cambridgeshire, UK) was dissolved in 100% ethanol, before being diluted in growth medium to the indicated concentrations. The CB1 antagonist SR141716A (Sanofi-Aventis, Paris, France) and the CB2 antagonist SR144528 (Sanofi-Aventis) were dissolved in DMSO and added so that the final concentrations of ethanol and DMSO were each 0.1% by volume. Cells were treated in the presence or absence of AEA (0.3–30 µM) and with or without CB1/CB2 antagonist (30 µM) for a maximum of 48 h, with photomicrographs taken at the beginning of treatment and after treatment using an inverted Nikon Eclipse TE2000-U phase-contrast microscope equipped with a Nikon DN-100 digital camera at 100× magnification, as described (Habayeb et al. 2008a). Total cellular RNA was prepared and RT-qPCR for histone deacetylase 3 (HDAC3), thyroid hormone receptor β 1 (TRβ1) and 5α reductase type 1 (5αR1) performed as described (Habayeb et al. 2008a; Khare et al. 2006) using the primers and annealing temperatures listed in Table 1.

### Statistical analyses

All statistical analyses were performed using GraphPad PRISM software (GraphPad PRISM v. 9:40; San Diego, CA, USA). The RT-qPCR data were subjected to Grubb’s test with *Q* = 1 and obvious outliers eliminated before D’Agostino–Pearson omnibus normality test was applied. Data were all normally distributed and so are presented as mean ± SD for the six samples in each group. One-way analysis of variance (ANOVA) (both Brown–Forsythe and Welch tests) with *α* set at 0.05 was used with Dunnett’s T3 multiple comparison test or Sidak’s multiple comparisons test used as appropriate for multigroup comparisons. The control group in all comparisons was preterm non-labouring women (PTNL). Correlation coefficients of these data were determined using Pearson’s linear correlation test and used to create a correlation matrix. Power analysis of similar data from mRNA and IHC studies showed that a sample size of *n* = 3 per group could offer statistically significant results (Ayakannu et al. 2015); nevertheless, we used a more conservative sample size of *n* = 6 to ensure statistically significant analyses (Taylor et al. 2009b).

## Results

### Plasma AEA concentrations

**Fig. 1:**
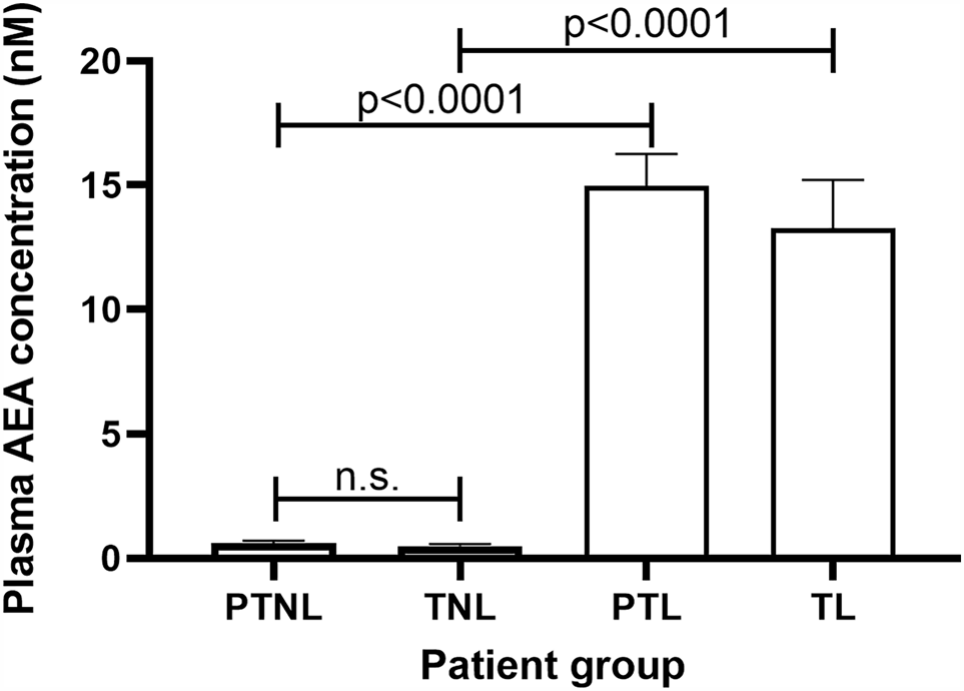
Effect of labour and prematurity on plasma AEA levels. Plasma obtained from each patient group indicated was subjected to UHPLC–MS/MS. Data are presented as the mean ± SD for six patient samples in each group, with each sample examined in triplicate. Differences in plasma concentrations was determined using one-way ANOVA with Dunnett’s T3 multiple comparisons tests; *p*-values are shown for significant comparisons; *n.s.* not significant, *PTNL* preterm not in labour, *TNL* term not in labour, *PTL* preterm not in labour, *TL *term in clinical labour

Plasma AEA concentrations in this patient cohort (Fig. 1) were very similar to those previously reported (Bachkangi et al. 2019b; Habayeb et al. 2004b; Nallendran et al. 2010). AEA concentrations were less than 1 nM in the non-labouring groups [preterm (PTNL) and term (TNL)]. The concentrations in the PTNL group were slightly higher than the term non-labouring group (TNL), but the difference was not statistically significant (*p* > 0.05). The values in those women in labour, whether preterm (PTL) or term (TL), were significantly higher (> 10 nM) than those of women in the non-labouring group (Fig. 1). The mean plasma AEA concentrations in the preterm group were slightly higher than those of the term group, but the difference was again not statistically significant (*p* > 0.05). These data are qualitatively and quantitatively similar to those previously reported (Bachkangi et al. 2019b).

### CB1 and CB2 receptor expression

**Fig. 2:**
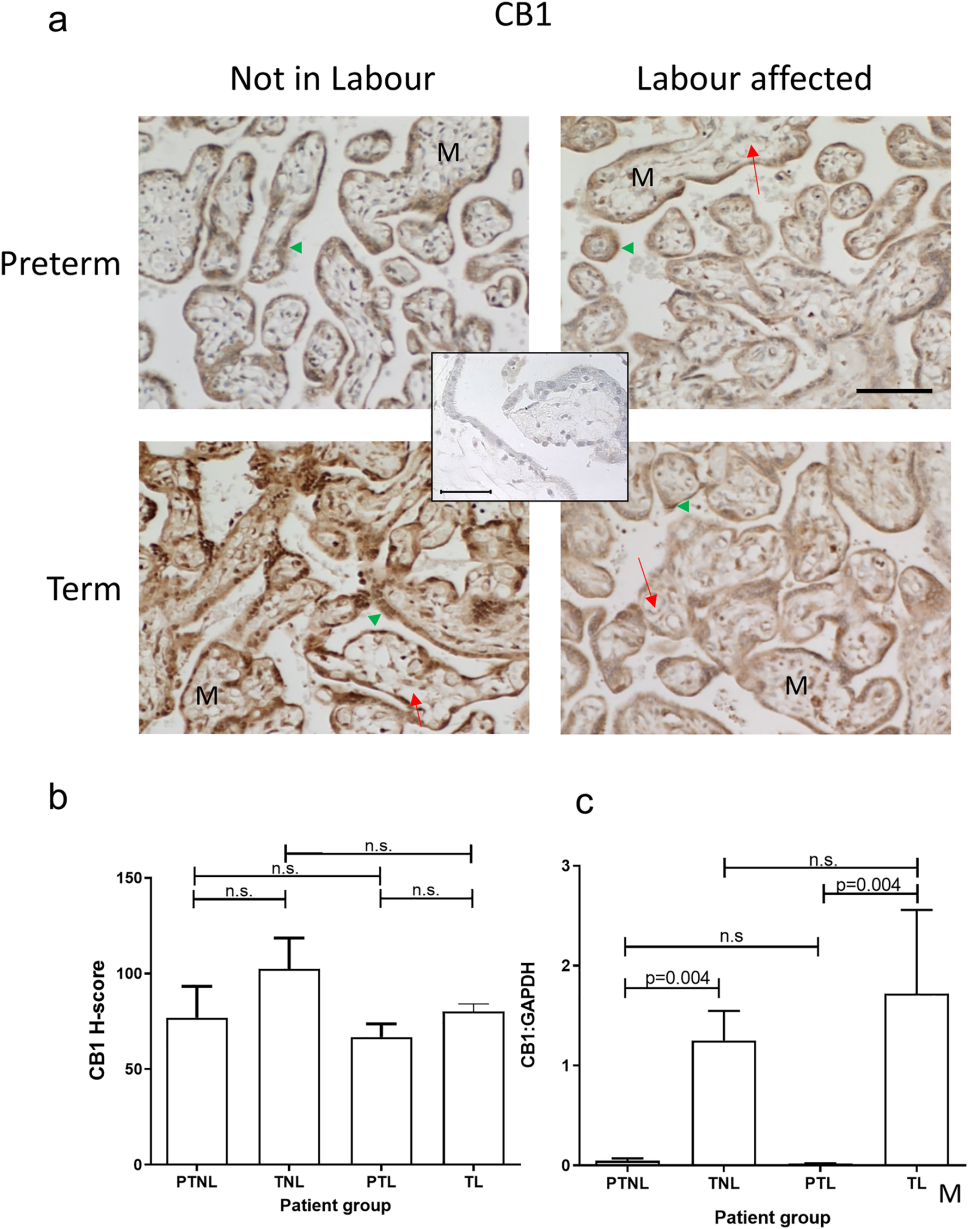
Effect of labour and prematurity on protein and CB1 transcript expression and levels. Representative photomicrographs of villous trophoblasts from ten images taken for each patient within each of the four patient groups (*n* = 6 samples per group) are shown in panel **a**. Images were obtained at 200× magnification; insert shows IgG negative control obtained at 40× magnification; bar, 50 μm. *M* indicates the mesenchymal core, whilst the green arrowhead points to the syncytiotrophoblast layer. Foetal capillaries within the mesenchymal cores are shown by the red arrows. Panel **b** shows the results of histomorphometry analyses of the 240 CB1 immunoreactivity images for the four different patient groups. Differences between groups were calculated using one-way ANOVA; *n.s.* non-significant. Panel **c** shows the levels of CB1 mRNA corrected for the levels of GAPDH. Data are presented as the mean ± SEM of six patient samples in each group, with each sample examined in triplicate. Differences in expression levels was determined using one-way ANOVA with Dunnett’s T3 multiple comparisons tests; *p*-values are shown for significant comparisons

Immunohistochemical staining for CB1 was limited mainly to the cytotrophoblast and syncytiotrophoblast (outermost) layers of placental villi (Fig. 2A). The staining for CB1 in placentae from both labouring groups was reduced slightly in comparison with that of the non-labouring groups, with CB1 staining highest in the term non-labouring group (TNL). Histomorphometric analysis indicated no significant changes (*p* > 0.05) in CB1 staining across all four groups (Fig. 2B), whereas placental levels of CB1 mRNA were significantly higher in both labouring groups when compared with those in placentae from women who were not in labour (Fig. 2C).

**Fig. 3:**
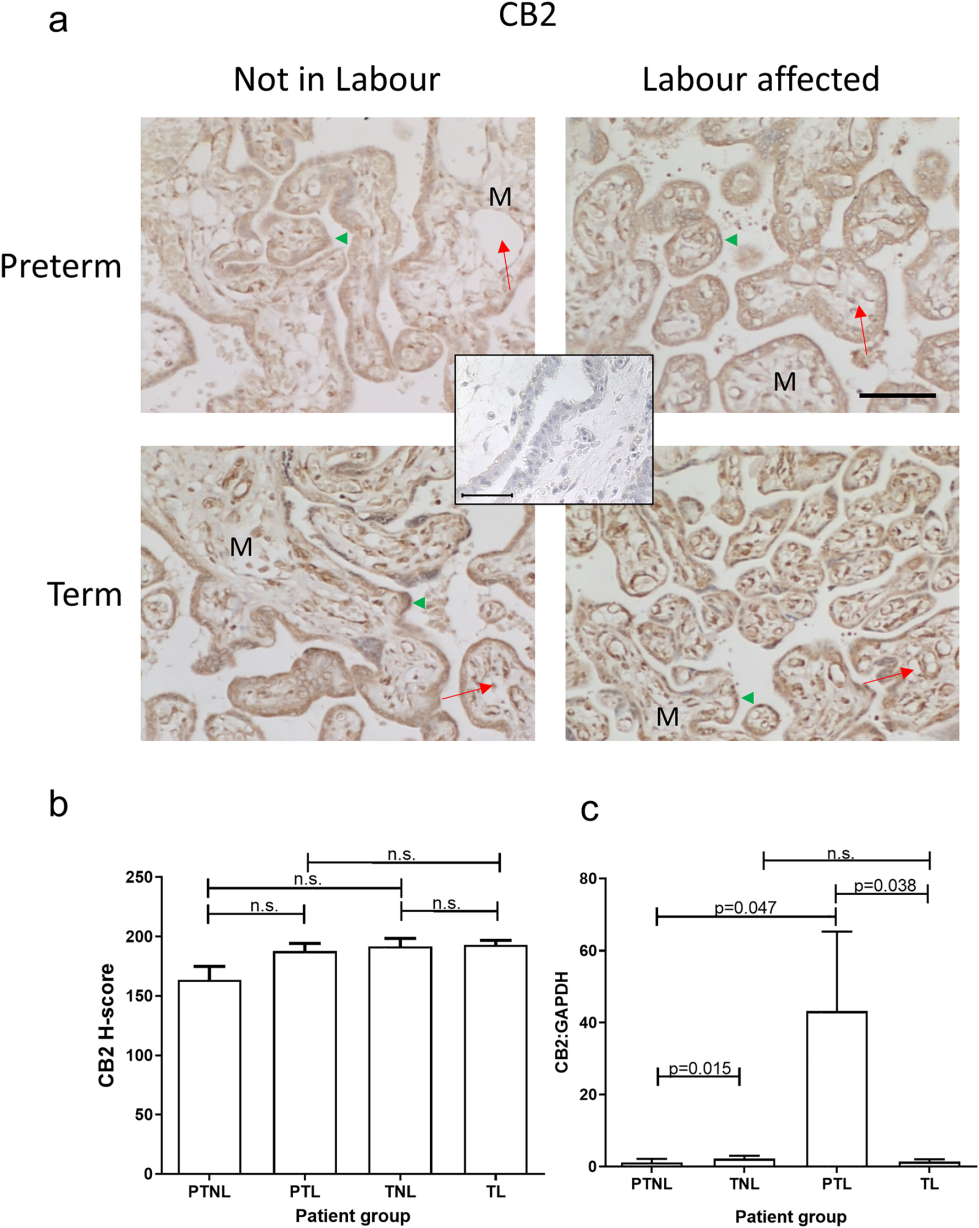
Effect of labour and prematurity on CB2 protein and transcript expression and levels. Representative photomicrographs of villous trophoblasts from ten images taken for each patient within each of the four patient groups (*n* = 6 samples per group) are shown in panel **a**. Images were obtained at 200× and 40× magnification as described in the legend to Fig. 2. Bar, 50 μm. Panel **b** shows the results of histomorphometric analyses of the 240 CB2 immunoreactivity images for the four different patient groups. Differences between groups were calculated using one-way ANOVA; *n.s.* non-significant. Panel **c** shows the levels of CB2 mRNA corrected for the levels of GAPDH. Data are presented as the mean ± SEM of six patient samples in each group, with each sample examined in triplicate. Differences in expression levels were determined using one-way ANOVA with Dunnett’s T3 multiple comparisons tests; *p*-values are shown for significant comparisons

Immunohistochemical staining for CB2 was also limited mainly to the cytotrophoblast and syncytiotrophoblast (outermost) layers of placental villi, although at term, CB2 staining increased in the foetal endothelial lining within the mesenchymal chore of the villi (Fig. 3A). The staining for CB2 in placentae from both labouring groups was slightly increased in comparison with both non-labouring groups, with CB2 staining highest in the term labouring group (TL). Histomorphometric analysis indicated no significant changes (*p* > 0.05) in CB2 staining across all four groups (Fig. 3B), whereas placental levels of CB2 mRNA were significantly higher in the term non-labouring group when compared with those from preterm non-labouring women (Fig. 3C). Interestingly, the pattern of CB2 mRNA expression was not identical in the preterm and term groups. In the transition from the non-labouring to labouring state, the expression of CB2 mRNA increased significantly in the preterm placentae but decreased significantly in term placentae (Fig. 3C). These data suggest that CB2 might be more important than CB1 in the transduction of maternal plasma AEA in the placenta at the end of pregnancy.

### NAPE-PLD and FAAH expression

**Fig. 4:**
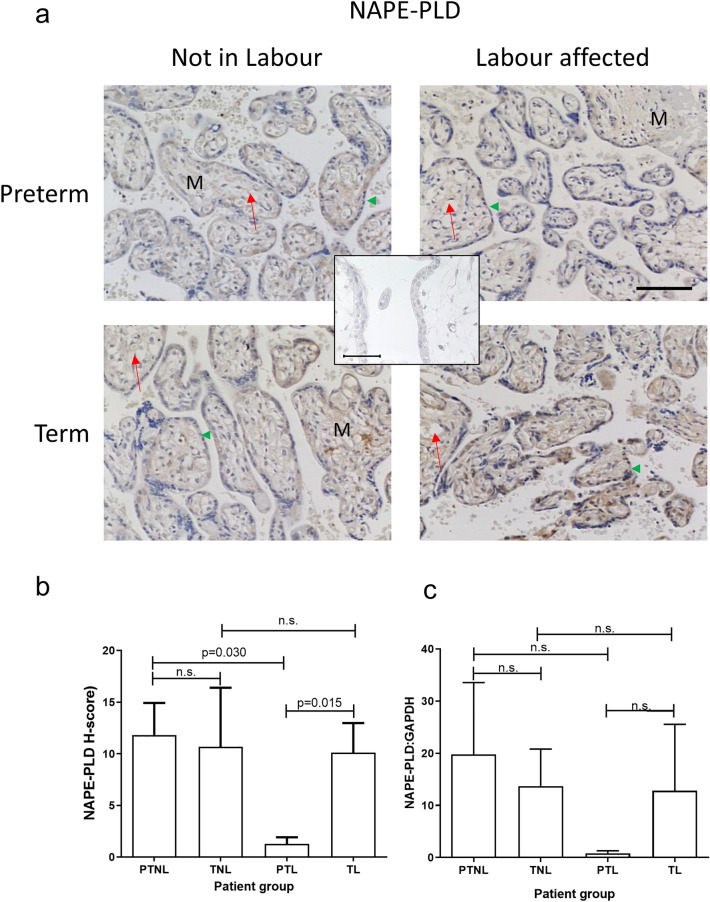
Effect of labour and prematurity on NAPE-PLD protein and transcript expression and levels. Representative photomicrographs of villous trophoblasts from ten images taken for each patient within each of the four patient groups (*n* = 6 samples per group) are shown in panel **a**. Images were obtained at 200× and 40× magnification as described in the legend to Fig. 2. Bar, 50 μm. Panel **b** shows the results of histomorphometric analyses of the 240 NAPE-PLD immunoreactivity images for the four different patient groups. Differences between groups were calculated using one-way ANOVA; *n.s.* non-significant. Panel **c** shows the levels of NAPE-PLD mRNA corrected for the levels of GAPDH. Data are presented as the mean ± SEM of six patient samples in each group, with each sample examined in triplicate. Differences in expression levels were determined using one-way ANOVA with Dunnett’s T3 multiple comparisons tests; *p*-values are shown for significant comparisons

Immunoreactive NAPE-PLD staining was very weak and observed only in the cytotrophoblast and syncytiotrophoblast layers just as it was for the immunostaining for CB1 and CB2 (Fig. 4A). No discernible staining was evident in the foetal endothelial cell lining or the mesenchymal core of the preterm placental villi, but was present in the term villi (Fig. 4A). Histomorphometric analyses (Fig. 4B) revealed similar NAPE-PLD protein levels in both non-labouring groups, and the term labouring group, but was significantly decreased in the pre-term labouring (PTL) group (Fig. 4B). Similarly, NAPE-PLD mRNA levels exhibited a similar pattern of expression as the protein in the placenta with a decreased level in the PTL group, although these data did not reach statistical significance (Fig. 4C). These data suggest that a decreased NAPE-PLD expression in preterm labour may be important.

**Fig. 5:**
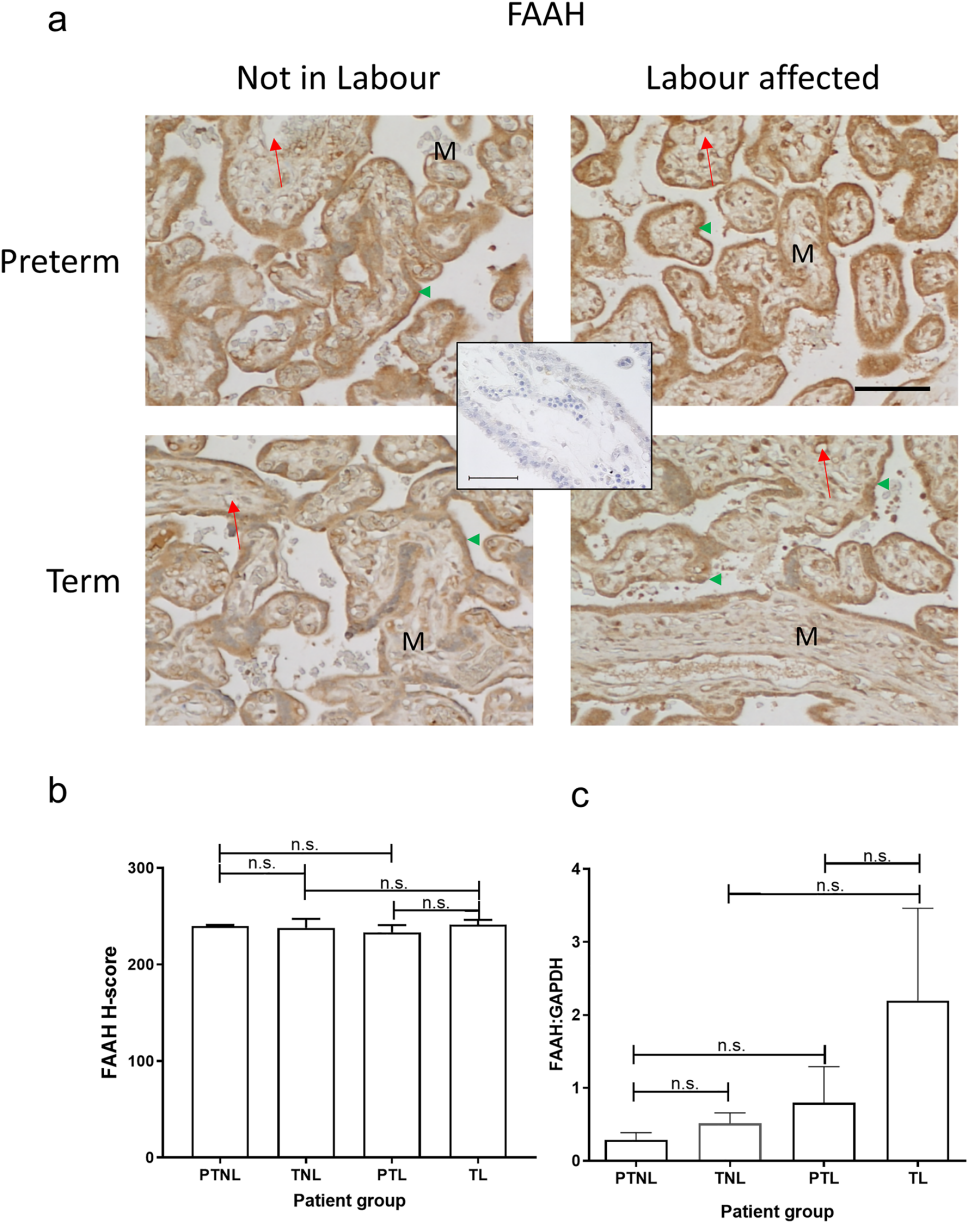
Effect of labour and prematurity on FAAH protein and transcript expression and levels. Representative photomicrographs of villous trophoblasts from ten images taken for each patient within each of the four patient groups (*n* = 6 samples per group) are shown in panel **a**. Images were obtained at 200× and 40× magnification as described in the legend to Fig. 2. Bar, 50 μm. Panel **b** shows the results of histomorphometric analyses of the 240 FAAH immunoreactivity images for the four different patient groups. Differences between groups were calculated using one-way ANOVA; *n.s.* non-significant. Panel **c** shows the levels of FAAH mRNA corrected for the levels of GAPDH. Data are presented as the mean ± SEM of six patient samples in each group, with each sample examined in triplicate. Differences in expression levels were determined using one-way ANOVA with Dunnett’s T3 multiple comparisons tests; *p*-values are shown for significant comparisons

Although FAAH immunoreactivity was detected throughout the placenta, including in the foetal endothelial cell layer of the mesenchymal core (Fig. 5A), the most intense staining was observed in the syncytiotrophoblast layer. Staining was slightly more intense in the preterm groups compared with the term groups, with no significant difference (*p* > 0.05) in FAAH expression among the four groups noted after histomorphometric analyses (Fig. 5B). FAAH mRNA levels showed a gradual increase in expression across the four groups; levels increased in placentae from both sets of labouring women when compared with the levels in non-labouring women (Fig. 5C), although because the data were variable, the differences observed were not statistically significant. Nevertheless, the increase in FAAH mRNA in term labouring patients (Fig. 5C) was qualitatively and quantitatively similar to that reported previously (Habayeb et al. 2004a).

### Correlation analyses

**Fig. 6:**
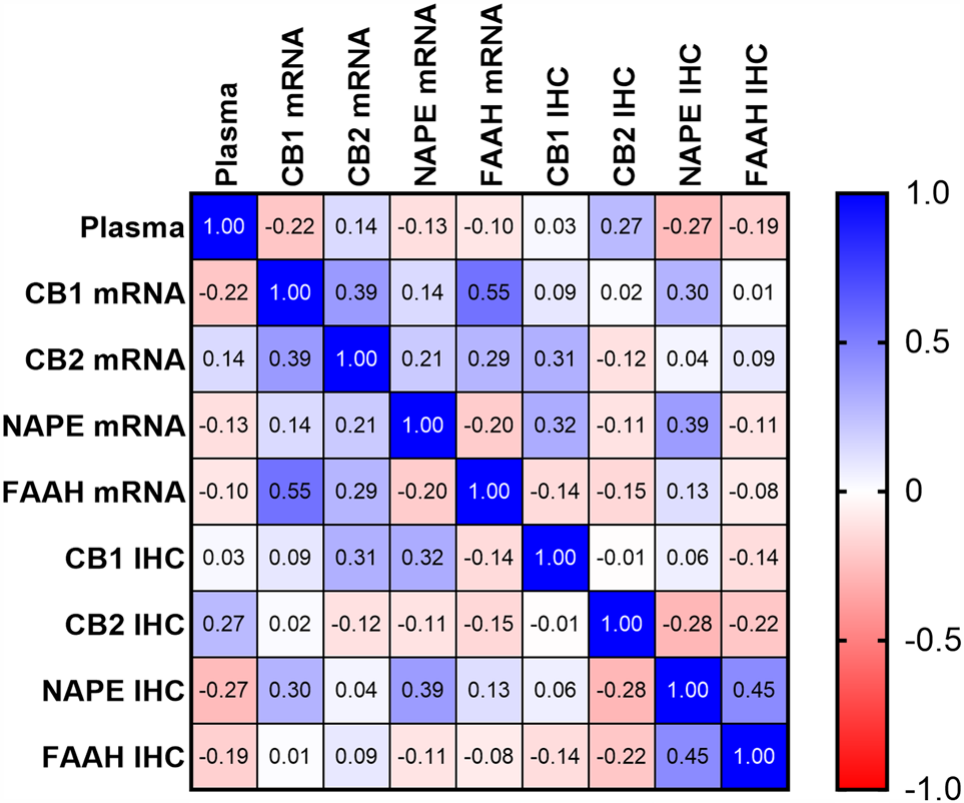
Correlation matrix of plasma AEA concentrations against the transcript (mRNA) and protein (IHC) levels for CB1, CB2, NAPE-PLD or FAAH. Potential relationships between plasma AEA concentrations (plasma), CB1, CB2, NAPE-PLD and FAAH mRNA or CB1, CB2, NAPE-PLD and FAAH H-scores (IHC) are shown with positive correlations depicted in blue and negative correlations depicted in red. The Spearman correlation coefficient (*r* values) are indicated. Statistically significant and non-significant correlations are presented in Table 2

To determine if any of the changes in the components of the ECS that occurred in the placenta at the end of pregnancy were related to each other, correlation analyses were performed (Table 2), and a correlation matrix was created (Fig. 6). The data indicated that changes in plasma AEA that occurred either in labour or during the end or pregnancy were not significantly correlated with mRNA or protein levels for CB1, CB2, NAPE-PLD or FAAH (Table 2; column 1). The analyses also revealed that only the mRNA and protein levels for NAPE-PLD were closely correlated (*r* = 0.39) across the entire cohort of 24 patients, whilst those for CB1, CB2 and FAAH indicated no such correlation. These data suggest that NAPE-PLD expression is important in the placenta towards the end of pregnancy and is tightly controlled. Other significant relationships were a positive correlation between FAAH and NAPE-PLD H-scores (*r* = 0.45), and CB1 and FAAH mRNA levels (*r* = 0.55), an observation previously reported for the respective proteins (Habayeb et al. 2008a). These data suggest functional differences in the expression of the ECS in the placenta at the end of pregnancy. It also suggests that any function of the CB2 receptor and its expression is not directly linked to other components of the ECS.

**Table 2:**
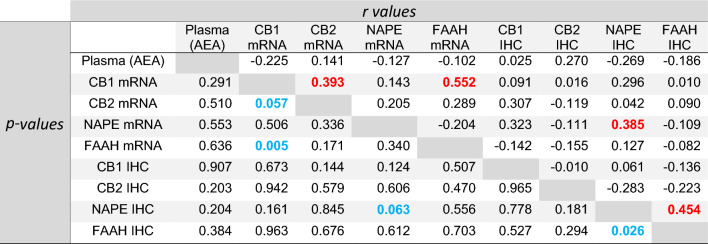
Correlation matrix

Significant correlation *r* values (top half of table) are shown in red, and significant *p*-values (lower half of table) in blue. Each parameter was independently assessed with *p* < 0.1 considered statistically significant; Spearman correlation analysis (*n* = 24)

### The effect of AEA on TCL-1 cells numbers

**Fig. 7:**
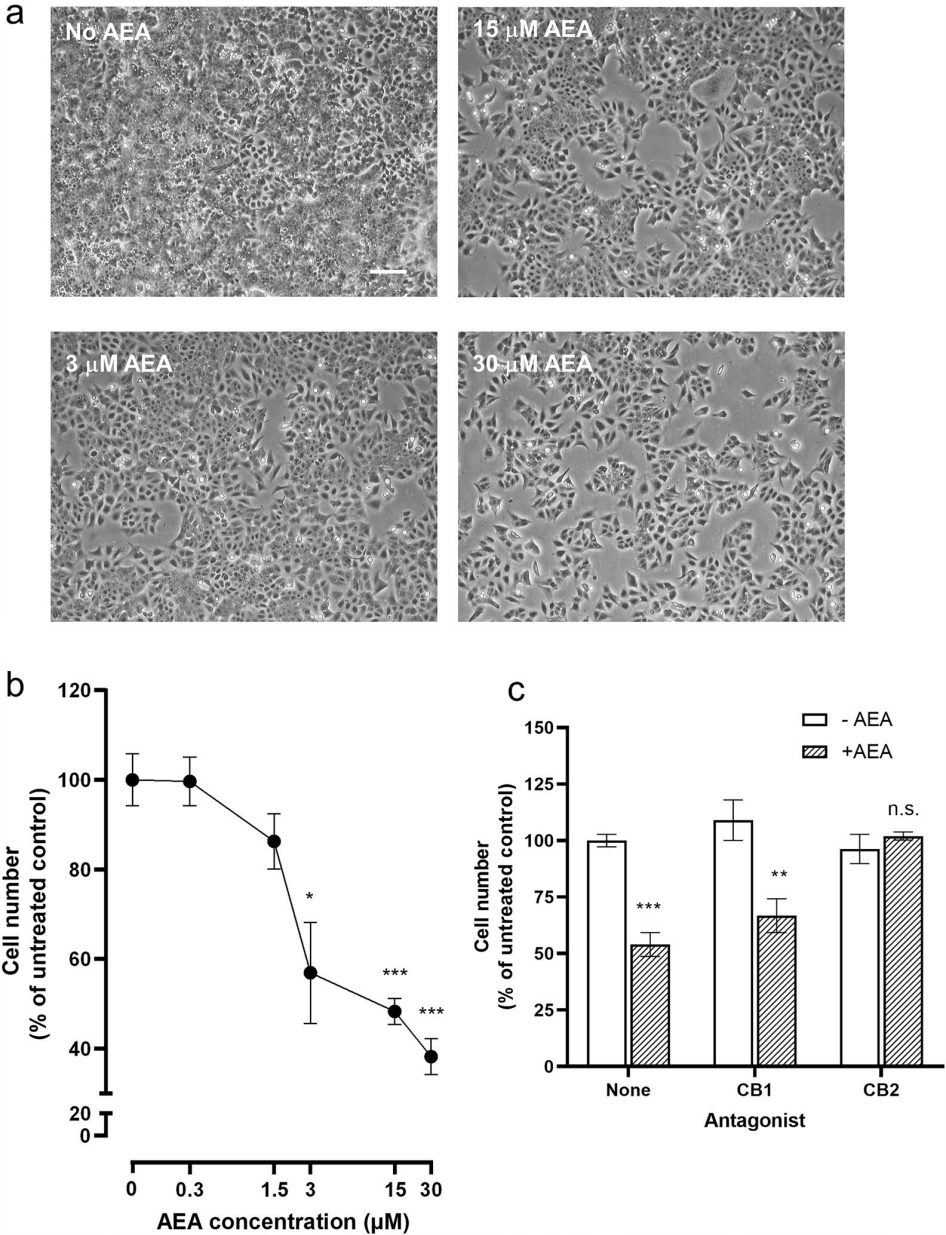
Effect of AEA on TCL-1 cell growth and survival. Representative photomicrographs of third-trimester trophoblasts (TCL-1 cells) grown in the indicated concentrations of AEA for 48 h are shown in panel **a**. Note the dose-dependent reduction in cell density. Each experiment was performed four times in triplicate. Bar, 120 µm. Panel **b** shows the results of the XTT proliferation and apoptosis assay. The data are presented as the mean ± SEM of four experiments performed in quadruplicate and relative to the untreated (control) cultures. *p < 0.05; **p < 0.01; ***p < 0.0001; one-way ANOVA with Dunnett’s T3 multiple comparisons tests. Panel **c** shows a similar XTT cell density experiment for TCL-1 cells treated with AEA (3 μM) in the presence of the CB1 antagonist SR141716a (CB1) or SR144528 (CB2). Data are presented as the mean ± SEM of three experiments performed in quadruplicate and relative to the untreated (control) cultures (−AEA; none). **p < 0.01; ***p < 0.0001; *n.s.* non-significant; one-way ANOVA with Dunnett’s T3 multiple comparisons tests

When sub-confluent cultures of the third trimester trophoblast cell line TCL-1 were treated with AEA, the cultures failed to reach confluence (Fig. 7A). Using the cell proliferation and apoptosis analysis (XTT) kit (Roche Diagnostics, Burgess Hill, West Sussex, UK), the reduction in cell density due to the AEA showed a gradual and significant effect above 3 µM AEA, with 30 µM AEA causing a maximal 60% reduction in cell numbers when compared with TCL-1 cell cultures not treated with AEA (Fig. 7B). To determine which of the CB receptors might be responsible for this effect, experiments were repeated with the minimal effective dose of AEA (3 µM) in the presence and absence of a CB1 or CB2 antagonist (Fig. 7C). The data showed that 3 µM AEA caused a 40% reduction in cell numbers when compared with the untreated control, and that the CB1 antagonist SR141716A had no effect, but the CB2 antagonist SR144528 abolished the AEA-induced reduction in TCL-1 cell numbers. These data suggest that the increased plasma AEA concentrations observed in labour (Fig. 1) affect trophoblast cell density in the placenta through the CB2 receptor.

### The effect of AEA on HDCA3, TRβ1 and 5α reductase 1 mRNA levels

**Fig. 8:**
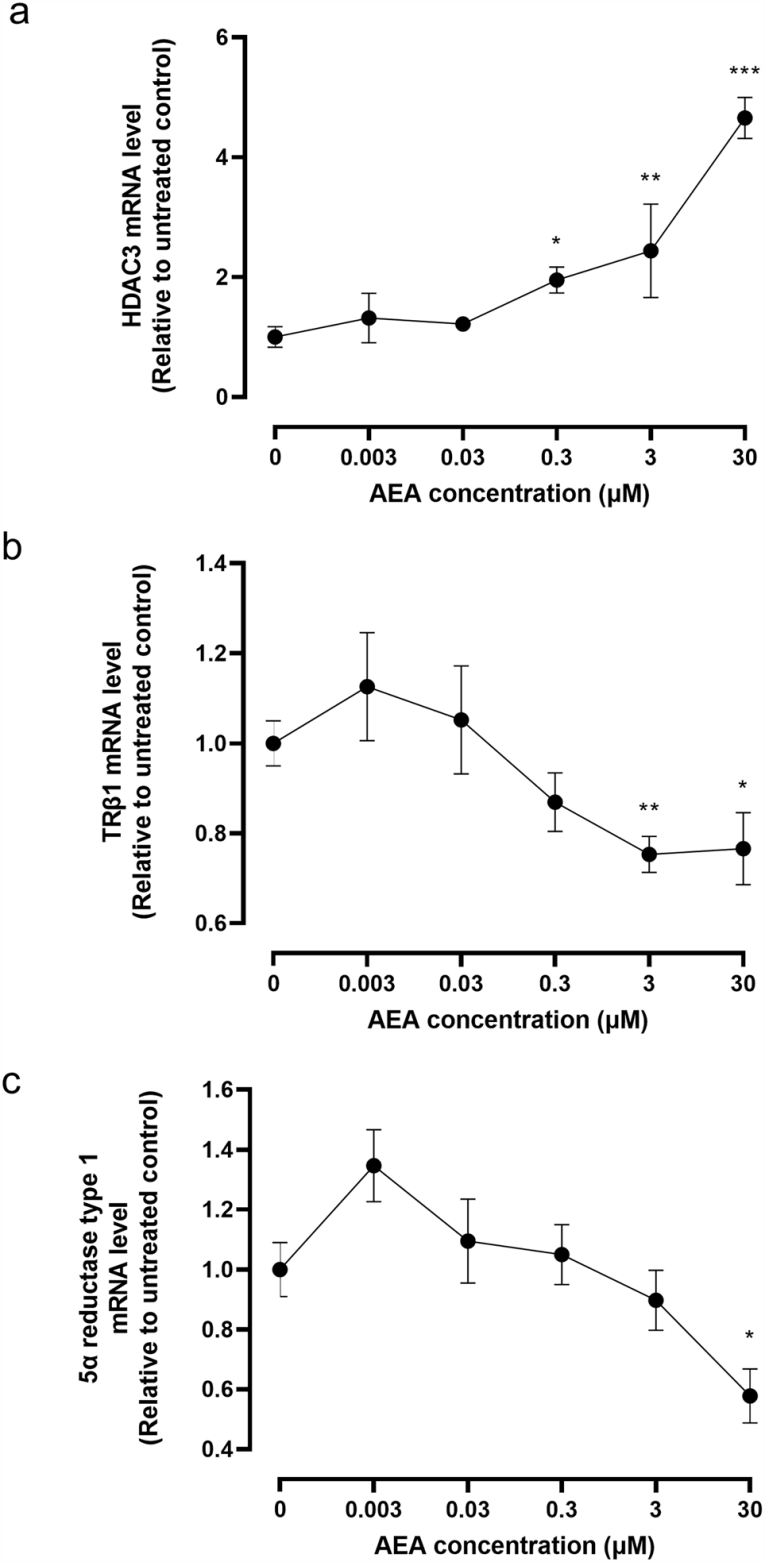
Effect of AEA on HDAC3, TRβ1 and 5α-reductase type 1 transcript levels in TCL-1 cells. Panel **a** shows the levels of HDAC3 mRNA, panel **b** the levels TRβ1 mRNA and panel **c** the levels of 5α-reductase type 1 mRNA generated with RT-qPCR corrected for the levels of GAPDH. Data are presented as the mean ± SEM of six samples in each group, with each sample examined in triplicate. Differences in expression levels was determined using one-way ANOVA with Dunnett’s T3 multiple comparisons tests; *p < 0.05; **p < 0.01; ***p < 0.0001

TCL-1 cells treated with AEA increased their expression of HDAC3 mRNA in a dose-dependent manner with a maximal effect of approximately four-fold with 30 µM **(**Fig. 8A). Simultaneously, AEA maximally decreased TRβ1 mRNA by 20% (Fig. 8B), and decreased 5αR1 mRNA by 40% (Fig. 8C). These data are similar to those obtained in the first-trimester trophoblast cell line BeWo treated with Δ^9^-THC (Khare et al. 2006).

## Discussion

The data presented herein confirm previous observations (Habayeb et al. 2004b; Marczylo et al. 2009; Nallendran et al. 2010) that plasma AEA concentrations increase in women that transition from the quiescent third-trimester state to term and then increase further when transitioning into labour, regardless of whether that labour state is at term or preterm. The data also support the conclusions of several studies that have shown that the ECS plays a role in all stages of human pregnancy, from early decidualisation to later stages where elevated plasma levels of AEA and other *N*-acylethanolamine ligands are positively associated with labour (Bachkangi et al. 2019a; Taylor et al. 2010b; Nallendran et al. 2010; Habayeb et al. 2004b, 2008a). Since the increasing AEA plasma concentrations might be a reflection of placental function, or as has been suggested, a source of prostaglandins required for labour (Maia et al. 2020b), we focused our attention on the expression of placental ECS during late normal pregnancy, and preterm and term labour (Bachkangi et al. 2019a) and found that transcripts and immunoreactive proteins for all components of the ECS [both cannabinoid receptor (CB1 and CB2), and both enzymes (NAPE-PLD and FAAH)] were present in the placentae of all the women (Figs. 2, 3, 4 and 5). Furthermore, we also found that NAPE-PLD mRNA and protein levels were closely linked (Fig. 4 and Table 2). These data suggest that the human placenta may be one source of the increased plasma AEA concentration levels observed during labour.

Interestingly, although plasma AEA concentrations were highest in the PTL group (Fig. 1), the same group had the lowest levels of NAPE-PLD protein and mRNA (Fig. 4). Similarly, there was no concomitant change in the expression of NAPE-PLD protein and mRNA in the placentae of women who delivered at term. These observations suggest that the increased plasma AEA concentrations seen during labour are not due to nascent production and release from the placenta, but could be from somewhere else in the body, an idea we proposed previously (Marczylo et al. 2009). These data are confirmed by the correlation analyses indicating that none of the placental ECS components showed a significant correlation with plasma AEA concentrations. By contrast, a recent study Maia et al. demonstrated that NAPE-PLD mRNA levels increase in the placentae of women with pre-eclampsia, whilst their plasma AEA, OEA and DHEA concentrations decreased. Those authors concluded that changes observed in ECS expression at the tissue level do not always mirror changes in plasma NAE levels (Maia et al. 2023). Our data tend to support that conclusion, especially as only one type of NAPE-PLD exists in mammals (Harrison et al. 2014). Maia et al. also showed increased FAAH expression in the placentae of women with pre-eclampsia, supporting a hypothesis that placental FAAH expression might be the main catabolic enzyme that regulates plasma AEA concentrations in such women. Indeed, we previously suggested that the increased expression of FAAH in the placenta during threatened and spontaneous miscarriage could be a protective mechanism against pregnancy loss when plasma AEA concentrations are on the rise (Habayeb et al. 2004b). At the end of pregnancy, the expression of FAAH might be expected to decrease in line with increased plasma AEA concentrations. This hypothesis is not supported by the data presented here, which showed no change in FAAH protein levels across all four groups (Fig. 5). Although there is a ‘hint’ of increased mRNA levels in both labouring groups, the differences were not statistically significant and are contrary to the hypothesis that placental FAAH would be diminished if it was responsible for the increased plasma AEA concentrations. From these observations, it is clear that placental FAAH expression and function is not important in the regulation of maternal plasma AEA concentrations at the end of pregnancy and that AEA must be produced elsewhere but is utilised locally to affect placental cell function. This also suggests that the foetus is not the source of the increased plasma AEA, since we have already discounted that option (Marczylo et al. 2009). This is because, to allow increased transport across the placenta from the foetus, placental FAAH expression would need to decrease, and that is obviously not the case here, as is suggested for women with pre-eclampsia (Maia et al. 2023), although supportive of a similar study performed with a small number of samples (Aban et al. 2013) where nitric oxide was the mediator, an effect we have previously reported during endometrial receptivity (Melford et al. 2021). FAAH expression in the placenta of all women was identical to that we and others reported previously (Park et al. 2003; Aban et al. 2013; Fonseca et al. 2012; Habayeb et al. 2004a; Kenney et al. 1999; Kozakiewicz et al. 2021; Maia et al. 2019), suggesting that FAAH still has an undiscovered role in the human placenta, even though FAAH knockout mice demonstrate increased tissue and plasma expression of all the NAEs (Kozakiewicz et al. 2021; Ortega-Gutierrez et al. 2004; Torella et al. 2019; Wang et al. 2008). Further studies would be needed to not only confirm out findings but perhaps unravel the basis of our suggestion that other enzymes may be important for NAE regulation in the placenta at term or during preterm labour.

The preceding observations suggest that local AEA production, might have effects on the placental tissue itself through its CB receptors, or through modulation of their expression patterns. The data indicated that mRNA levels of CB1 increased as pregnancy approaches term and remained elevated when women were in labour (Fig. 2). By contrast, a similar increase in the women who had a preterm labour showed no increase in CB1 mRNA. The pattern of CB1 protein expression in the cohort mirrored that of the mRNA levels, but the differences were not statistically significant regardless of whether that delivery occurred at term or preterm. The IHC staining patterns were identical to those reported previously (Lombo et al. 2022; Park et al. 2003; Fonseca et al. 2012; Habayeb et al. 2008a; Taylor et al. 2011a), with CB1 receptors on the endothelial cells of the mesenchymal core and the cytotrophoblast/syncytiotrophoblast layer on the surface of the maternal interface (Fig. 2). The presence of the CB1 receptor at these sites may be an indicator of AEA effects on angiogenesis or vasculogenesis, as previously reported (Maia et al. 2022; Taylor et al. 2011b) or may alter vascular plasticity (Baranowska-Kuczko et al. 2012; Domenicali et al. 2005). This latter effect may be important in pre-eclampsia (Lombo et al. 2022).

Alternatively, we hypothesised that AEA could be affecting the placental cells directly through the activation of trophoblast cell survival. The data obtained using the third-trimester cell line, TCL-1 (Figs. 7 and 8), suggest that AEA does indeed affect trophoblast cell proliferation or survival, but not through the CB1 receptor, since the presence of a CB1 receptor antagonist did not abrogate the inhibitory effect of AEA on cell numbers (Fig. 7). The levels of CB1 receptor mRNA were significantly lower in the preterm groups when compared with their term counterparts (Fig. 2), suggesting that AEA would not be active in the placenta of such women; however, as is shown in Fig. 2, the protein levels remained at reasonable (but somewhat variable) levels through the entire cohort. The observation that CB1 expression and mRNA levels were lower in women presenting in preterm labour suggests that the expression of these receptors is important in maintaining a functional placenta until term. Indeed, we and others have examined the expression and function of the CB1 receptors in first-trimester placentae and reported that their loss is associated with early pregnancy loss (Fonseca et al. 2012; Habayeb et al. 2008a, 2008b; Karasu et al. 2014; Park et al. 2003; Taylor et al. 2007, 2011a; Trabucco et al. 2009), so loss of the CB1 receptors in preterm placentae is unsurprising and thus indicative of a failing placenta.

Furthermore, we found that the CB2 expression at the mRNA and protein levels had a significant reduction in CB2 transcript levels which were associated with PTNL, but with no corresponding changes in protein expression in the tissues (Fig. 3). The increase in placental CB2 mRNA levels in PTL samples was variable but statistically significant, suggesting that CB2 gene activation could be important in the aetiopathogenesis of PTB (Habayeb et al. 2008a). The observation that the AEA-induced reduction of TCL-1 cell numbers could be abrogated by the presence of the CB2 antagonist SR144528 (Fig. 7) indicates that AEA has a growth-inhibitory effect on term placenta and its stability directly through the CB2 receptor. The majority of the term placenta are syncytiotrophoblast, generated from the fusion of earlier trophoblast cells. Nevertheless, the syncytiotrophoblast needs to be maintained during this critical stage of pregnancy if the foetus is to be delivered alive. In this regard, the remaining cytotrophoblast becomes a critical cell, because loss of these cells could destabilise the placenta and thus contribute to the onset of labour. The differential expression of HDAC3 in TCL-1 cells when compared with our previous data on its expression in AEA-treated BeWo cells points to the use of different receptors in the two cell lines with AEA activating the CB1 receptor in BeWo cells and AEA activating the CB2 receptor in TCL-1 cells. Changes in HDAC3 expression have been associated with the formation of syncytin, a vital protein involved in syncytiotrophoblast formation and stability (Chuang et al. 2006). This is especially important towards the end of pregnancy because a differential trophoblast/syncytiotrophoblast ratio may be key in initiating labour (Firestein et al. 2022). The HDAC3 increase in response to AEA could be related to a different role of HDAC3 and other histone deacetylases in the increased regulation of some cytokines (Munro et al. 2021) that are known to affect the activation of the myometrium in preterm deliveries, especially interleukin 1 (Nadeau-Vallee et al. 2017). Stability and correct functioning of the placenta is also dependent on thyroid hormones, which in turn need appropriate expression of their receptors, such as TRα1 and TRβ1 (Kilby et al. 1998; Ohara et al. 2004). Failure of these receptors often results in foetal neural deficits or demise (Adu-Gyamfi et al. 2020; Ohara et al. 2004). Additionally, the foetus is exquisitely sensitive to androgen excess especially in female offspring (Vu et al. 2009), an effect that is negated by increased 5αR1 in males (Mahendroo et al. 1997). In this regard, the increase in plasma AEA concentrations in preterm pregnancies may be protective of the foetus whereby the decreased expression of critical genes that prevent foetal demise (TRβ1and 5αR1) leads to physiological changes that induce early foetal delivery, perhaps through increased HDAC3 expression and downstream cytokine activities. These data suggest that the placental endocannabinoid system may be linked to the initiation or progression of preterm labour and that downstream events associated with altered CB receptor expression and function in the placenta need further investigation.

## Conclusions

At the end of pregnancy, the placental ECS appears to play a key role in controlling the trophoblast/cytotrophoblast/syncytiotrophoblast structure and function, especially during labour. The main effect of AEA in the placenta is to reduce trophoblast cell numbers through CB2 and thus contributes to the appropriate stability of the placenta during labour with concomitant changes elsewhere at the foetal–maternal interface. The dramatic changes in general regulators of transcription point towards increased cell damage at this critical point at the end of pregnancy.

## Data Availability

All data are available in the main text.
